# Physical performance reference values for Japanese oldest old: a SONIC study

**DOI:** 10.1186/s12877-022-03299-7

**Published:** 2022-09-13

**Authors:** Kiyoaki Matsumoto, Yasuyuki Gondo, Yukie Masui, Saori Yasumoto, Yuko Yoshida, Kazunori Ikebe, Yasumichi Arai, Mai Kabayama, Kei Kamide, Hiroshi Akasaka, Tatsuro Ishizaki

**Affiliations:** 1 Graduate School of Human Sciences, Osaka University, Osaka, Japan; 2 Tokyo Metropolitan Institute of Gerontology, Tokyo, Japan; 3 Department of Prosthodontics, Gerodontology and Oral Rehabilitation, Osaka University Graduate School of Dentistry, Osaka, Japan; 4 Center for Supercentenarian Medical Research, Keio University School of Medicine, Tokyo, Japan; 5 Division of Health Sciences, Osaka University, Graduate School of Medicine, Osaka, Japan; 6 Department of Geriatric and General Medicine, Osaka University Graduate School of Medicine, Osaka, Japan

**Keywords:** Aging, Physical performance, Oldest old, Reference value, Japanese older adults, SPPB

## Abstract

**Background:**

The oldest old, defined as those aged 90 or over, is now the fastest-growing population sector. This study aimed to determine reference values for several physical performance measures (PPMs) among 90-year-olds using internationally standardized measurements and to clarify the characteristics of these indices by comparing their results for 90-year-olds with those for older people 70 and 80.

**Methods:**

We used the Septuagenarians, Octogenarians, and Nonagenarians Investigation with Centenarians (SONIC) study data from 2010 to 2018. The study subjects were 70, 80, and 90-year-olds in the target area eligible to participate in the venue. Excluding those certified for long-term care, the final number of eligible persons is 70s cohort 1000 (2010), 80s cohort 973 (2011), and 90s cohort 690. 90s cohort only consisted of three survey waves: 2012, 2015, and 2018. We used hand grip strength and score on the Short Physical Performance Battery (SPPB) for our physical performance measurements. In addition, we statistically analyzed sex and age differences.

**Result:**

The simple mean ± standard deviation (SD) for the 90-year-old respondents were in men, 24.1 ± 5.4 kg in hand grip strength, 0.80 ± 0.22 m/s in usual gait speed, 17.2 ± 6.73 s in 5times chair stand, 5.89 ± 4.42 s in tandem balance, and 8.3 ± 2.2 in SPPB respectively and in women, 14.4 ± 4.0 kg in hand grip strength, 0.72 ± 0.20 m/s in usual gait speed, 17.8 ± 7.89 s in 5times chair stand, 4.72 ± 4.35 s in tandem balance, and 7.5 ± 2.4 in SPPB, respectively. For all PPMs, the age 90 cohort was statistically significantly different from the age 70 and 80 cohorts (all trends *P* <  0.001). Hand grip strength decreased with a similar gradient with age cohort increase of 10 years for both sexes. In contrast, SPPB lower limb score showed a larger drop between the age 80 and 90 cohorts than between the age 70 and 80 cohorts. We also constructed sex-specific appraisal standards according to quintiles.

**Conclusions:**

Our study yielded inclusive sex-specific reference values and appraisal standards for major physical performance measures not certified as requiring long-term care, community-dwelling, oldest old Japanese. The characteristics of age-related decline in physical performance differed between the upper and lower extremity assessments.

## Background

The decline in physical performance with aging is a critical issue for older adults because such declines limit older citizens’ activities of daily living (ADLs) and instrumental activities of daily living (IADLs). These limitations in turn inhibit independence and autonomy, which decreases overall quality of life. In particular, in the population of the oldest old, it takes them a great deal longer to recover from illness and fracture than it does younger people [1], so it is important to detect declines in physical performance early and offer appropriate interventions, such as exercise and therapy.

In the United States and Japan, where the aging populations have grown, age 90 years or older has been proposed as the defining age for the oldest old. The defined age is based on the increase in the number of oldest old people and their current status [2, 3]. In developed countries with aging societies, the number of residents in both countries who are over age 90 is rapidly increasing, and this trend is expected to continue and spread to developing countries [4].

In previous surveys of the oldest old, researchers have used various methods to assess their physical performance [5–8]. Some researchers have used objective measurements such as gait speed and hand grip strength that are used with the younger elderly to evaluate the oldest old, and some measure functioning based on survey scores related to IADL performance [9]. Many if not most studies of the oldest old have been conducted in Europe and the United States [5–8, 10–12], and because the subjects of these studies were Europeans, who are taller and heavier than Japanese and other Asians, it is difficult to use them as a reference for comparison with Asians. Therefore, the ﻿Asian Working Group for Sarcopenia (AWGS) advocated establishing separate cutoff values that take into account the body size of Asians from the EUROPE criteria [13] and establishing cutoffs for physical performance that match the current status of aging in each country [14, 15]. In addition to the above research deficiencies, other researchers tend to bundle their results for nonagenarians (people in their 90s) with results for study participants aged 80 or 85 or older [16]. Even in studies of people aged 90 and older, researchers do not make comparisons with other age groups or examine their characteristics, and only some report on physical performance indicators in 90-year-olds [17–20]. These various gaps in the literature mean that there are no published reference values for physical performance measures (PPMs) in the age group of 90 and above, which manifests in a few ways. For instance, most researchers assume that the oldest old are frail or weak [14, 21, 22], but some could set unduly high goals for this group and recommend excessive exercise and treatment. Without effective reference values, there is no accumulation of evidence on preventive and therapeutic interventions for the growing population of the oldest old.

The Georgia Centenarian Study compared centenarians in Tokyo and Georgia and found that those in Tokyo had lower physical functioning than those in Georgia, with the researchers identifying differences in medical care, living conditions, disabilities, and health status between the two countries as reasons for the differences in functioning [23]. Further examining the subject will require evaluating the physical functioning in the oldest members of society including those aged 90. As the latter group continues to increase in populations worldwide, establishing reference points for functioning in the oldest old will be critical for assessing whether pathological physical declines occur.

According to Cress et al., the state of physical function in the oldest old is highly variable [22]. Therefore, to properly assess the physical function of the oldest old, combining multiple indicators that take into account the ceiling or floor effect of the outcome is desirable, rather than just a single physical function assessment. In their life course approach to healthy aging, Kuh et al. stated that age-related changes in objective indicators and their association with other constructs are significant [24]. They advocate the need for longitudinal cohort studies using standardized measures to examine such associations. However, no studies in Japan have adequately assessed the physical functions of the oldest old using internationally established physical assessment methods and examined the results.

### Objectives of this study

Based on the above, the first objective of this study was to propose reference PPMs using internationally comparable physical function assessment methods specifically for individuals aged 90 or above, who traditionally have been grouped with younger elderly populations. The second objective was to compare the reference values for age 90 with those for ages 70 and 80 and to clarify the differences and characteristics of this age group from other age groups of older adults.

## Methods

### Participants

The participants in this study were community-dwelling, independently living people who participated in a study of health and longevity called the Septuagenarians, Octogenarians, Nonagenarians Investigation with Centenarians (SONIC), an ongoing prospective cohort study of older people as part of the Centenarian Study initiated in June 2010 [25]. The SONIC study aims to assess the characteristics of a general population sample of older people consisting of three control groups of different ages (70s, 80s, and 90s) for a centenarian cohort and to clarify the factors for health, longevity, and psychological well-being. We conducted this study in two main regions of Japan, eastern and western (Tokyo metropolitan area and Hyogo Prefecture, respectively). Both regions include urban and rural areas.

The data collection for each age cohort was performed in different years because of a large number of participants recruited. The participants were recruited from residential registries and contacted by postal mail.

The SONIC study used a narrow age-range cohort design. Inclusion criteria were older adults whose ages fall within a range of 3 years of each cohort (70 80 90). The first-wave survey began in 2010 for the 70s cohort, 2011 for the 80s cohort, and 2012 for the 90s cohort. Each cohort includes individuals whose ages fall within a range of 3 years (e.g., 69–71 for a 70-year-old cohort). Due to the small number of participants only in the 90s cohort, participants of the 90s were newly recruited and added to the 90s cohort in the second (2015) and the third (2018) waves.

In the first wave (2010 ~ 2012), there were no restrictions on the conditions for participation. However, in the second (2015) and the third (2018) waves, the conditions for participation were set to exclude those who were certified for requiring long-term care or could not walk independently without assistance.

Of the 4307 eligible participants in the 70s cohort, 3307 who did not consent to an on-site survey were excluded, resulting in 1000 on-site participants in the 70s cohort (479 men and 521 women). Of the 5391 eligible participants in the 80s cohort, 4418 who did not consent to the on-site survey were excluded, resulting in 973 on-site participants in the 80s cohort (391 men and 410 women). Of the 4134 eligible participants in the 90s cohort, 3327 who did not consent to the on-site survey were excluded, resulting in 807 on-site participants in the 90s cohort (385 men and 404 women).

**Fig. 1:**
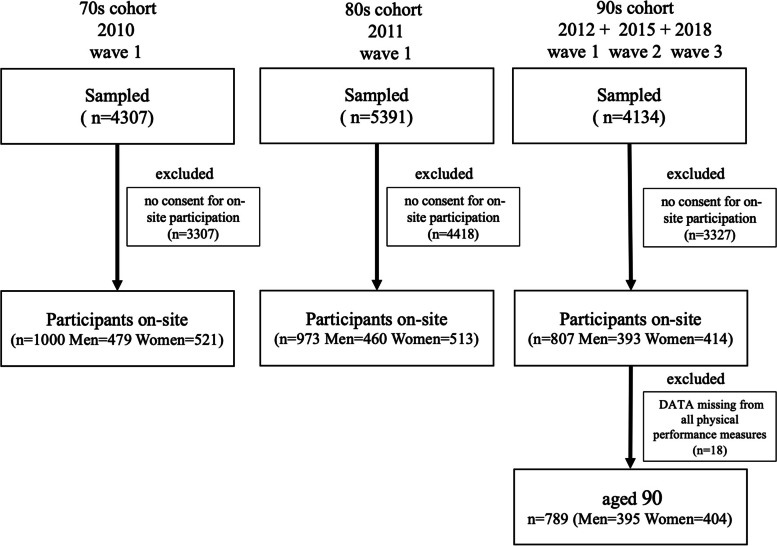
Flow chart of the details of the study participant selection

We used the 90s cohort (88–91), the oldest old, as the primary target group of this study. Of the 807 on-site participants aged 90 years, we excluded 18 who could not complete all physical function tests, leaving 789 participants from the different survey waves: 264 in 2012 (119 men and 145 women), 264 in 2015 (131 men and 133 women), and 261 in 2018 (135 men and 126 women). After we excluded 89 people who had been certified as requiring long-term care and 10 who were missing care insurance certification data, we were left with 690 study participants (344 males and 346 females) to establish physical performance reference values. Figure 1 presents a flow chart of the details of study participant selection.

### Methods for assessing physical performance

Building on earlier research challenges and limitations, we measured physical performance in a population aged 90 and above using scores for hand grip strength measured by a Smedley YD-100 hand grip dynamometer (Yagami Ltd., Tokyo, Japan) and the SPPB as the basis. Since these two methods have been used in many previous studies of the oldest old, the results of this study can be comparable to those of previous studies. Researchers have used a wide mix of items to evaluate physical performance in older adults [26–28]. Among the measures, researchers in Europe and the United States have used the SPPB to evaluate physical performance in frail elderly populations [29, 30]. Even the oldest old can perform it safely, and it has been validated to predict a wide range of indicators such as mortality and nursing care status [31, 32]. In addition, hand grip strength and gait speed are widely used measures for evaluating physical performance regardless of region or age, including in evaluating sarcopenia, and frail in older adults [18, 27, 29]. Researchers have used hand grip strength, in particular, to evaluate muscle strength even in age groups other than the elderly; it has been shown to be the most predictive and valid indicator for all-cause mortality [33].

### SPPB

The SPPB measures balance, gait, strength, and endurance on a scale of 0 to 12. A higher total SPPB score indicates higher lower limb function. Researchers use SPPB as one of the screening tools for functional status, such as sarcopenia [34]. Balance is measured as standing balance holding time with the feet aligned horizontally, semi-vertically (semi-tandem), and vertically (tandem). For this study, we measured gait following Guralnik’s original SPPB gait measurement method [29]. We used a static start method in which we measured usual gait speed from the starting line within a frame of 8 ft. (2.44 m) without an acceleration period. Lower limb strength including endurance is assessed by measuring the time from sitting in a chair to standing for five repetitions. For the chair stand test, we used a chair at the Japanese standard height of 40 cm. We followed the original SPPB scoring criteria [29].

### Hand grip strength

In this study, we used the measurement method recommended by the American Society of Hand Therapists [35], whereby participants were required to sit, rotate their shoulders inward to a neutral position, bend their elbows to 90°, place their forearms in a neutral position, and dorsiflex their wrists between 0° and 30°. We measured hand grip strength twice with the participant’s dominant hand up to 1/10th of a kilogram. If the participant could not use the dominant hand to grasp, whether because of a fracture or for other reasons, we used the non-dominant one. For maximum hand grip strength, we used the stronger of the two measurements.

### Assessing health-related information

We measured height and weight at the site and calculated body mass index (kg/m^2^). We collected other information, medical history, self-rated health, and drinking and smoking history, using self-administered questionnaires.

### ADLs and IADLs

There are two methods for evaluating functioning in the elderly. Basic ADLs include walking, moving, eating, and bathing, and higher-order IADLs include managing transportation, meal preparation, medication, and finance. We used the Barthel Index [36], which is a widely used measure for assessment and rehabilitation, to assess the study participants’ basic ADL functioning. The Barthel Index scale allows evaluation of functional independence in 10 ADLs (feeding, grooming, bathing, dressing, bowel and bladder care, toilet use, and mobility [ambulation, transfers, and stair climbing]). The responses of the 10-item BI are scored on five levels ranging from 0 to a maximum of 5, 10, or 15, with a total score of 100.

To measure IADLs, we used the Tokyo Metropolitan Institute of Gerontology Index of Competence (TMIG-IC). Lawton systematized seven activity skill levels required for the elderly to live independently in the community. Based on those skills, Koyano et al. developed the TMIG-IC; now, it is widely used for measuring IADLs in Japan [37]. There are 13 yes-no questions with 1 point for each yes, for a score range of 0 to 13 points; higher scores indicate higher functioning.

### Statistical analysis

We used descriptive statistics to characterize the study population by means and standard deviations. We analyzed differences between men and women using the unpaired T-test, chi-squared test, and Mann-Whitney U test and calculated PPM means and standard deviations (SDs) for all 90-year-olds and each sex. To compare linear trends in the PPM means within the age cohort, we used one-way analyses of variance by sex. We used quintiles of each PPM to construct appraisal standards according to sex for age 90, which are criteria for determining whether the decline in physical function in the oldest old in question is a standard decline due to aging or a pathological decline. We set significance at *p* < .05 and performed all statistical analyses using IBM SPSS Statistics version 25.

## Result

Table 1 summarizes the characteristics of the 90-year-old study participants. All PPMs were significantly higher in men than women, except for the 5times chair stand test. Men had significantly higher ADL scores than women, but there were no statistically significant differences between men and women on the IADLs.

**Table 1:** Characteristics of all study participants

Variables	Mean ± standard deviation or n (%)		*p*
	Men (*n* = 1324)	Women (*n* = 1438)	
Age cohort, n (%)			
70	479 (47.9)	521 (52.1)	
80	460 (47.3)	513 (52.7)	
90	385 (48.8)	404 (51.2)	
aged 90	Men (*n* = 385)	Women (*n* = 404)	
recruit year			
2012	119 (45.1)	145 (54.9)	
2015	131 (49.6)	133 (50.4)	
2018	135 (51.7)	126 (48.3)	
Geographic area, n (%)			
Urban			
Itami	127 (50.8)	123 (49.2)	
Itabashi	132 (42.0)	182 (58.0)	
Rural			
Asago	77 (55.0)	63 (45.0)	
Okutama	49 (57.6)	36 (42.4)	
Height, cm	158.8 (6.1)	144.9 (6.1)	< 0.001
Weight, kg	55.7 (8.3)	45.8 (7.4)	< 0.001
long-term care insurance			
certification, n (%)	36 (9.4)	53 (13.3)	0.087
Body mass index, kg/m2	22.1 (2.9)	21.8 (3.2)	0.207
Chronic disease, n (%)			
Hypertension	249 (65.0)	282 (70.0)	0.137
Dementia	15 (4.1)	19 (5.1)	0.807
Stroke	45 (11.7)	30 (7.5)	0.083
Parkinson’s disease	2 (0.5)	1 (0.2)	0.535
Heart disease	133 (34.5)	120 (29.7)	0.145
diabetes *mellitus*	58 (15.1)	51 (12.7)	0.320
Self-rated health, n (%)			
Excellent to good	304 (79.2)	328 (81.8)	0.362
Fair to poor	80 (20.8)	73 (18.2)	
Alcohol drinking status, n (%)			
Current	179 (45.5)	45 (10.7)	< 0.001
Past	68 (25.3)	25 (14.0)	< 0.001
Smoking status, n (%)			
Current	31 (7.8)	10 (2.4)	< 0.001
Past	279 (71.5)	32 (7.7)	< 0.001
Barthel Index (ADL)	96.47 ± 8.80	93.36 ± 12.23	< 0.001
TMIG-IC (IADL)	9.94 ± 3.03	9.35 ± 3.61	0.155
Physical performance measures			
Hand grip strength, kg	23.7 ± 5.69	14.1 ± 4.13	< 0.001
Usual gait speed, m/s	0.78 ± 0.22	0.70 ± 0.21	< 0.001
Tandem balance, sec	5.7 ± 4.45	4.3 ± 4.33	< 0.001
5times chair stand, sec	17.1 ± 6.83	17.4 ± 6.27	0.156
SPPB total score	8.2 ± 2.33	7.3 ± 2.51	< 0.001

*ADL* Activities of Daily Living, *TMIG-IC* the Tokyo Metropolitan Institute of Gerontology Index of Competence, *IADL* Instrumental Activities of Daily Living*, SPPB* the Short Physical Performance Battery

Table 2 shows the number of participants unable to perform each PPM and those who had difficulty with the five-times sit-to-stand test, which took more than 20 s. The percentages of unusable data were 2.1% (*n* = 17 participants), 6.7% (*n* = 17), 19.7% (*n* = 158), and 6.6% (*n* = 53) for hand grip strength, usual gait speed, sit-to-stand test, and tandem balance, respectively. The number of people who took more than 20 s to stand up from a chair five times was 168, or 20.8% of the total. The lowest and highest rates of missing data were for hand grip strength and the 5times chair stand test, respectively.

**Table 2:** Numbers of participants with unable data for each variable

Variables	age	70		80		90	
		unable, (%)	difficulty,(%)	unable, (%)	difficulty,(%)	unable, (%)	difficulty,(%)
Physical performance measures							
Hand grip strength		16 (1.6)	–	5 (0.5)	–	17 (2.1)	–
Usual gait speed		15 (1.5)	–	14 (1.4)	–	54 (6.7)	–
Tandem balance		2 (0.2)	–	12 (1.2)	–	53 (6.6)	–
5times chair stand		16 (1.6)	1 ^a^(0.1)	46 (4.7)	32 ^a^(3.3)	158 (19.7)	168 ^a^(20.9)

^a^5times chair stand > 20 sec

Tables 3 and 4 present unweighted simple PPM means and SDs according to age group in men and women, respectively. For all PPMs, the age 90 cohort was statistically significantly different from the age 70 and 80 cohorts (all trends *P* <  0.001). In women, all PPMs showed a significant decreasing trend as the age cohort increased. In contrast, there were no statistically significant differences in 5times chair stand, tandem balance, or SPPB total score between male 70- and 80-year-olds.

**Table 3:** Descriptive statistics for physical performance measures according to age group (men)

					*p*
Variables	Mean ± standard deviation				for trend
	Overall	Age group			
		70	80	90	
Hand grip strength, kg (n)	28.8 ± 6.9 (1259)	32.9 ± 6.6 (471)	28.1 ± 5.7 (444)	24.1 ± 5.4 (344)	< 0.001
Usual Gait speed, m/s (n)	0.90 ± 0.22 (1250)	0.96 ± 0.23 (471)	0.91 ± 0.20 (414)	0.80 ± 0.22 (336)	< 0.001
5times chair stand, sec (n)	13.53 ± 5.0 (1217)	12.2 ± 3.32 (468)	12.3 ± 3.47 (436)	17.2 ± 6.73 (313)	< 0.001
Tandem balance, sec (n)	8.26 ± 3.5 (1255)	9.39 ± 2.19 (478)	9.26 ± 2.31 (442)	5.89 ± 4.42 (335)	< 0.001
SPPB total score (n)	10.1 ± 2.0 (1250)	10.8 ± 1.4 (471)	10.6 ± 1.6 (443)	8.3 ± 2.2 (336)	< 0.001

*SPPB* the Short Physical Performance Battery

**Table 4:** Descriptive statistics for physical performance measures according to age group (women)

					*p*
Variables	Mean ± standard deviation				for trend
	Overall	Age group			
		70	80	90	
Hand grip strength, kg (n)	17.5 ± 4.9 (1358)	19.9 ± 4.8 (513)	17.1 ± 4.3 (499)	14.4 ± 4.0 (346)	< 0.001
Usual Gait speed, m/s (n)	0.88 ± 0.23 (1343)	0.99 ± 0.22 (514)	0.88 ± 0.21 (495)	0.72 ± 0.20 (334)	< 0.001
5times chair stand, sec (n)	13.35 ± 5.4 (1263)	11.7 ± 3.46 (516)	12.6 ± 3.56 (472)	17.4 ± 6.27 (273)	< 0.001
Tandem balance, sec (n)	7.53 ± 3.97 (1348)	9.20 ± 2.46 (521)	8.23 ± 3.45 (496)	4.72 ± 4.35 (331)	< 0.001
SPPB total score (n)	9.77 ± 2.3 (1346)	10.9 ± 1.3 (516)	10.1 ± 1.9 (497)	7.5 ± 2.4 (333)	< 0.001

*SPPB* the Short Physical Performance Battery

Hand grip strength decreased equitably with a similar slope (value of the difference between ages 70–80 and 80–90: M 4.79, 4.03; W 2.84, 2.67) with an increasing 10-year age cohort for both sexes. In contrast, usual gait speed, tandem balance, and SPPB total score (value of the difference each between ages 70–80 and 80–90: M 0.18, 2.26; W 0.76, 2.57) showed a larger drop between the 80–90 age cohort than the drop between the 70–80 age cohort. Finally, Table 5 shows the PPM quintiles for men and women of age 90, excluding those who were certified as requiring long-term care. We calculated means for each physical function assessment by sex for the 90-year-old subjects, used these means as reference values, and constructed sex-specific evaluation criteria according to the quintiles. In addition, hand grip strength and SPPB total score show ADL and IADL values according to each quintile (Table 5). Although we found a ceiling effect in tandem balance in men, all other PPMs had an approximately symmetrical distribution. The means and SDs for each PPMs were as follows: Hand grip strength 24.1 ± 5.4 kg, usual gait speed 0.80 ± 0.22 m/s, five-times chair stand 17.2 ± 6.73 s, tandem balance 5.89 ± 4.42 s, and SPPB 8.3 ± 2.2 respectively, in men and hand grip strength 14.4 ± 4.0 kg, usual gait speed 0.72 ± 0.20 m/s, five-times chair stand 17.8 ± 7.89 s, tandem balance 4.72 ± 4.35 s, and SPPB 7.5 ± 2.4, respectively, in women. Both ADLs and IADLs decreased at quintile 1 (Lowest) in hand grip strength. In SPPB, Both ADLs and IADLs showed a stepwise decrease from quintile 3 (Normal) to 1. All of these declines were statistically significant (*P* <  0.001).

**Table 5:** Quintiles of physical performance measures for 90 years old

Physical performance measures	Quintile levels		Men				Women			
Hand grip strength (kg)			mean	*n* = 344	ADL	IADL	mean	*n* = 346	ADL	IADL
	5	(Highest)		**28.0 < =**	98.0	10.3		**17.5 < =**	96.9	11.2
	4			25.0–27.9	97.3	11.0		15.5–17.4	96.1	10.2
	3		**24.1**	22.5–24.9	98.3	10.6	**14.4**	13.0–15.4	96.6	10.6
	2			19.5–22.4	98.8	11.1		11.0–12.9	96.0	11.2
	1	(Lowest)		**< 19.5**	**96.8**	**9.7**		**< 11.0**	**93.3**	**8.9**
Usual gait speed (m/s)				*n* = 336				*n* = 334		
	5	(Highest)		**1.00 < =**				**0.88 < =**		
	4			0.83–0.99				0.77–0.87		
	3		**0.80**	0.72–0.82			**0.72**	0.68–0.76		
	2			0.63–0.71				0.54–0.67		
	1	(Lowest)		**< 0.63**				**< 0.54**		
Tandem balance (s)				*n* = 335				*n* = 331		
	5	(Highest)		**10 < =**				**10 < =**		
	4			10 < =				7.2–9.9		
	3		**5.9**	4.0–10.0			**4.7**	1.9–7.1		
	2			0.1–3.9				0.1–1.8		
	1	(Lowest)		**0**				**0**		
5times chair stand (s)				*n* = 313				*n* = 273		
	5	(Highest)		**<= 12.3**				**<= 12.7**		
	4			14.6–12.4				15.0–12.8		
	3		**17.2**	17.0–14.7			**17.4**	18.0–15.1		
	2			21.0–17.1				21.9–18.1		
	1	(Lowest)		**21.0 <**				**21.9 <**		
SPPB total score				*n* = 336				*n* = 333		
	5	(Highest)		**10 < =**	98.8	11.1		**10 < =**	98.0	11.8
	4			9	98.9	11.0		8–9	97.7	10.9
	3		**8.3**	8	**98.6**	**10.5**	**7.5**	7	**96.6**	**10.1**
	2			7	**96.5**	**9.5**		6	**93.9**	**9.3**
	1	(Lowest)		**<= 6**	**94.7**	**9.0**		**<= 5**	**90.8**	**8.8**

*ADL* Activities of Daily Living, *IADL* Instrumental Activities of Daily Living, *SPPB* the Short Physical Performance Battery

## Discussion

### Main findings

The results of this study showed that hand grip strength decreased at a similar slope in each age group as the age group increased. In contrast, the total SPPB score, an indicator of lower limb muscle strength, showed a greater drop between ages 80 and 90 than between ages 70 and 80. This more significant drop suggests a rapid decline in physical function, especially in the lower limbs, during the transition from age 80 to age 90, the age of the oldest old.

Hand grip strength indicates a decline in total muscle strength, which gradually declines with aging. On the other hand, SPPB scores include not only muscle strength of the lower limbs but also the state of functional aspects such as gait and balance in the assessments. Therefore, it is possible that the SPPB is also influenced by the frequency of daily activities (standing up, moving). The rapid decline in lower limb function at age 90 is assumed to be due to much less outdoor mobility, such as a decrease in going out and participation in organizations.

In Japan, the SPPB has not been actively utilized in assessing older adults. The reason for this is that the original SPPB scoring cannot assess differences in lower limb function due to the ceiling effect, which often results in a perfect score, especially when older adults living in the community are targeted [38]. The results of this study showed that the means of the 70- and 80-year-olds were not normally distributed, with each score exceeding 10 and being close to the perfect score. In contrast, the SPPB at age 90 was normally distributed. Below the SPPB quintile three or lower, both ADL and IADL scores show a statistically significant decrease. This significant decrease suggests that at the age of 90, the decline in daily living function may be inferred by the SPPB value. Therefore, we suggest that SPPB is suitable for assessing the oldest old in Japan.

Table 6 presents a comparison of the findings from earlier large-scale cohort studies of physical functioning in elderly populations. These researches follow; the BFC80+ [39], the Leiden 85-Plus [40], the Tokyo Metropolitan Institute of Gerontology-Longitudinal Interdisciplinary Study on Aging (TMIG-LISA) 6 cohort study [16], the Newcastle 85-Plus Study [41], the BFC80+, the 90+ Study [17], the validity 90+ Study [18], the Danish 1905 cohort survey [19], and Genetics of Healthy Aging (GEHA) [20]. In the following sections, we use these previous studies to compare and discuss the results of this study.

**Table 6:** Assessment of physical performance in previous studies of the oldest old (85 years and older)

	85 to 90 years old								90 years old over									
Reserch name	The BFC 80+		The Liden 85-plus		TMIG-LISA 6 cohort study		The Newcastle 85-plus Study		The 90 + Study		Vaitality 90 + study		The Danish 1905 cohort survey 1998		GEHA		Present study	
Country	Belgium		Netherland		Japan		UK		USA		Finland		Denmark		Italia		Japan	
Age	85		85-89		85 over		85-90		94		90		92-93		90-94		90	
Sex Men (n) Women (n)	M (211)	W (356)	M (102)	W (255)	M (48)	W (68)	M (106)	W (188)	M (173)	W (456)	M (65)	W (197)	M (491)	W (1307)	M (369)	W (791)	M (344)	W(346)
Hand grip strength (kg)	M 30.6	W 17.8	M 25.6	W 16.4	M 23.2	W 15.2	M 21.05	W 11.5	M 20.3	W 11.1^d^	M 46	W 28^e^	M 22.8	W 13.4	M 22.0	W 13.5	M 24.1	W 14.4
Gait speed (m/s)	M 0.7	W 0.5^a^	ALL 0.52^a^		M 1.11	W 0.92^c^	−		M 0.66	W 0.52^d^	−^f^		M 0.64	W 0.52^g^	−^f^		M 0.8	W 0.72
5 times Chair stand (sec)	−		−		−		−		M 16.2	W 16.8^d^	M 18.0	W 20.0	−		−		M 17.2	W 17.8
SPPB	M 8.4	W 6.3^b^	−		−		−		modified SPPB		−		−		−		M 8.3	W 7.5^h^

*SPPB* the Short Physical Performance Battery

^a^Max gait speed

^b^Modified SPPB

^c^Gate speed with acceleration phase

^d^Values in the middle quartile

^e^Median value

^f^Walking ability (mobility) was interviewed by questionnaire

^g^Usual gate speed

^h^The original SPPB evaluation was used

### Possible causes for value differences

When comparing the results of different studies evaluating physical functions, it is important to use the same measurement methods. The differences between our results and those of previous studies might be attributed to the differences in measurement methods. In the following sections, we discuss possible causes for differences between studies in the evaluated PPMs and propose research directions for future studies focusing on the oldest old.

### Hand grip strength

In this study, hand grip strength decreased as age increased for both men and women, which was consistent with earlier findings among the elderly in Japan [42]. Compared to previous studies conducted in Europe, we had a higher value than in the Newcastle 85-Plus Study and lower than in the Leiden 85-Plus Study. These variations reflect the inconsistency in research findings on the oldest old age 90 and over. Hand grip strength varies depending on standing versus sitting, upper limb position, and forearm posture, so appropriate comparisons require similar measurement methods. In populations other than solely older adults, hand grip strength in Japan is often measured in a standing position with the elbow extended. There are few measurement data on older adults in a sitting position [43].

In examinations of differences in hand grip strength by posture, researchers found greater hand grip strength in the standing position than in people who were seated [35]. In previous studies, some researchers measured grip while standing, and some did not mention the measurement posture. These differences in measurement methods could have affected the results of comparing studies.

It is often difficult for oldest old people over age 90 to hold a standing position. In fact, about 20% of the 90-year-old participants in the SONIC study needed some kind of assistance to stand up and hold the standing position, and it was difficult to measure their ability to stand up from a chair. Not only the oldest old but also those who cannot hold a standing position are expected to have weaker grip (muscle) strength than those who can hold a standing position without holding anything. Therefore, a participant’s ability to hold the standing position is a prerequisite for measuring hand grip strength, which can cause selection bias; studies might show high findings because the authors selected people with high muscle strength. Similarly, some investigators measured hand grip strength in an unstable state in which the subject held the balance with one hand while standing. In the Leiden 85-Plus, where the hand grip strength was greater than in the present study, 14.8% of the subjects could not be measured, and it is possible that those results were influenced by selection bias because people who could not stably hold the standing position were excluded from the measurement.

In short, the effect of measurement position on 90-year-olds is significant due to their physical vulnerability, and therefore, when comparing PPMs, it is important to consider the measurement method (such as measurement position and frequency) more strictly than is generally necessary with older adults. The oldest old often have difficulty maintaining a standing position and are at greater risk of falling, which suggests that it is safer and more stable to measure hand grip strength of the oldest old in a sitting posture.

### Gait speed

This is the first study to report walking speed by gender in Japanese subjects aged 90 years who were not certified as needing long-term care using an internationally standardized measurement method. Researchers in Japan commonly use a dynamic start with an acceleration period before the measurement distance, whereas Western researchers commonly use a static start without an acceleration period. The dynamic start is faster because it reduces the influence of the slow acceleration period. Because studies on Japanese older adults, including the TMIG-LISA 6 cohort study, used dynamic start [16], it is not possible to accurately compare Japanese findings with findings from overseas studies. However, with the present study, we used the static start method, and thus, we consider that our findings are comparable with those from Western studies.

For the usual gait speed per second in this study, the rates for men (0.8 m/s) and women (0.72) were faster than the overall gait speeds in the Leiden 85-Plus (all: 0.52 m/s) [40] and BFC80+ [39] (age 85; M: 0.7 m/s, W: 0.5 m/s), which included participants at a younger average age than the subjects in the present study. The usual gait speeds we found were faster than those in the 90+ Study [17] (ages 90–94; M: 0.66, W: 0.52), which used older participants than ours, and those in the Danish 1905 cohort survey (ages 92–93; M: 0.64, W: 0.52), whose participants were also older.

The Leiden 85-Plus (age 89) and BFC80+ (age 85), both study cohorts were younger than the SONICs, measured maximum gait speed, while the other studies (90+ Study, Danish 1905) measured usual gait speed. Maximum gait speed is generally faster than usual speed. Our findings for usual gait speed were faster than the maximum gait speeds in other studies, indicating a faster gait speed in the oldest Japanese individuals than the speed of the oldest old in the West.

Research suggests that differences in mobility, including walking speed, are attributed to differences in lifestyle [44, 45]. Usual gait speed is highly sensitive in predicting ADL disability in people over 75 years old [43]. In the case of this study, we assumed that the differences in walking speed from findings in other studies were attributable to those people in Japan who tend to live a tatami mat lifestyle.; Japanese houses still have tatami flooring. In Japan, some older adults live on tatami mats. If older adults live in a room with tatami flooring, they sit on the tatami for meals and sleep in the tatami lifestyle. They regularly stand up and sit down, strengthening their lower limbs daily. It is necessary to investigate whether similar cultural factors and lifestyle features affect ADLs and other measurements of functioning in the oldest old in different countries.

In addition, 90-year-olds are often unable to participate in surveys outside homes due to their declining physical functioning. We adopted a gait distance of 2.44 m in this study because we considered that the oldest old citizens who were in institutions or could not come to the survey site could measure their gaits at the places where they lived. Since the advent of the COVID-19 pandemic in 2020, group surveys conducted in large venues have been severely restricted to prevent infection, and such restrictions are expected to continue in the future. Because the SPPB can be measured at home or in a facility with limited space, we considered it appropriate for measuring physical function in 90-year-olds even during the pandemic.

### Chair stand and chair height

The time in this study for the five-times sit-and-stand test (M: 15.9 and W: 16.3 × the upper limit of quartile 3 compared with the 90+ Study) was faster than those in the validity 90+ Study (M: 18.0, W: 20.0) for the same age group and those in the 90+ Study (M: 16.1, W: 16.7). In addition, the differences between men and women in the results of this study were small, and there was no statistically significant gender difference in the results in the 90-year-old reference value (3rd normal quintile). In addition, women in the SONIC study were faster in the 70-year-old age cohort.

It is possible that the chair’s height had an advantage for Japanese women, who are shorter than those in Europe and the United States and men. One of the factors that affect standing behavior is the height of the chair used for measurement [46]. In the SONIC study, a Japanese standard chair height of 40 cm was used for both men and women, and because women are shorter than men, a chair of the same height would make it relatively easier for women to stand up and harder for men. This relative ease of standing could be why there was little difference between men and women on the 5times chair stand test.

It is also possible that the differences in chair heights used between the studies in Europe, the United States, and Japan could have affected the rise times in this study in addition to the differences. However, we could not verify this proposition because researchers in the previous studies did not include the heights of the chairs used. Therefore, accurately comparing rising data internationally requires noting and considering differences in the chair heights used.

### Chair standing itself as a screening criterion

In this study, the proportion of participants who failed or were unable to perform the five-times sit-and-stand test was the largest among the tests of physical performance assessment: 1.6% in the age 70 cohort, 4.7% in age 80, and about 20% in age 90. In other words, the percentage of 90-year-olds who were unable to perform the test five times was much higher than that of other age cohorts.

Furthermore, compared between PPMs, the proportion of 90-year-olds who could not complete the chair stand test failure (19.7%) was larger than those for hand grip strength (2.1%) and normal gait speed (6.7%) among the other tests. In addition, 20.9% of the subjects took longer than 20 s to perform the test (Table 1). This means that at the age of 90, the test of standing up from a chair proves to be remarkably difficult.

In the Danish cohort survey of nonagenarians, 61% of men and 50% of women were able to stand up without using their hands [19]; in other words, 39% of men and half of women could not stand up without using their hands. Similarly, in the 90+ Study, 32% of all participants failed to complete the five-times sit-and-stand test. These results indicate significantly higher proportions of people who have difficulty standing up without using their hands in the oldest old (90 years and older) than in younger old age groups.

Guralnik originally gave an SPPB score of 0 for failures to complete the five-times sit-stand and a score of 1 to the lowest 25% of those who completed the test [29]. Based on the results of this study and of previous studies, it is possible that about one-quarter of the oldest old are unable to rise from a chair without holding on to something, which corresponds to the lower approximately 25% of the total oldest old population to the criterion for 1 point on the original SPPB. Therefore, the five-times sit-to-stand test could be a screening test for lower limb muscle strength. Indeed, the AWGS, in its 2019 criteria for the diagnosis of sarcopenia, allows general practices and facilities to use the test if they do not have dual-energy X-ray absorption or bioelectrical impedance analysis [15].

Researchers in a study of older adults in Japan used a test called the Frail 10-Second Chair Stand Test to measure the number of times a person could stand up in 10 s without using their hands in a similar way to the SPPB and found that it was independently associated with quadriceps strength and TUG test score, which represents dynamic balance [47]. These findings suggest that the chair stand test can be used to assess overall lower extremity muscle strength even when used alone in the oldest old over 90 years of age (in Japan).

### Study strengths and limitations

#### Study characteristics

The first distinguishing feature of this study is that we conducted it with older adults in Asia. Asian countries other than Japan are in the transition stage to aging societies, and the numbers of the oldest old are small and of little interest. Our study assessed the physical functional status of the oldest old within Japan specifically for the first time. The second distinguishing feature of our study is that we treat age 90 as a discrete group, not just as members of groups age 80 or 85 and above. As we have seen, there are currently no reference values for assessing the physical performance of nonagenarians. With this background, we included 90-year-olds as an independent age cohort.

The main strength of this study is that we had access to a large sample size of the oldest old over 90 years of age one country in Asia, where there are still few oldest old people; generally, there are fewer oldest old men than women, but rates in this study were similar: 48.8% for men and 51.2% for women. Furthermore, the results of this study can be easily compared with other studies because we assessed physical function using internationally standardized measurement methods. At present, there are no representative data for Asia’s oldest old, including in Japan, and which is why we believe our results can be used as the best current reference values for the oldest old people in Asian countries.

The main limitation of this study was selection bias. The subjects in this study were 90-year-olds who were not certified as needing long-term care who were otherwise eligible to participate. However, according to the 2015 census, approximately 50% of Japanese aged 90 and older are certified as needing long-term care in an institution [48]. Therefore, we assume that the participants in this study were relatively healthy community-dwelling 90-year-olds. The distribution of physical functions across all 90-year-olds can be estimated by adding assessments that include those certified as requiring long-term care. In addition, as mentioned in the discussion, we grouped the three populations from different participation years, so it is necessary to consider the rejuvenation phenomenon separately among these populations. Finally, due to the cross-sectional design of this study, it was impossible to make causal inferences about age or sex differences in PPMs.

## Conclusions

This study analyzed sex-specific reference values and appraisal standards for five PPMs in nondisabled, community-dwelling, Japanese oldest old (age 90 and over). This study indicates that a rapid physical performance decline occurs in the lower limbs during the transition from age 80 to age 90. Furthermore, this study indicates that the SPPB and the 5times chair stand test are practical physical function assessment methods that can be utilized in Japanese oldest old. Although absolute physical performance varies among populations, the age and sex differences in PPMs could be standard across Asian countries. The characteristics of these results in PPMs can be broadly shared with Japan and Asian countries.

## Data Availability

The datasets used and/or analyzed during the current study are available from the corresponding author on reasonable request.

## Abbreviations

AWGS: The Asian Working Group for Sarcopenia
ADL: Activities of Daily Living
IADL: Instrumental Activities of Daily Living
GEHA: Genetics of Healthy Aging
PPMs: Physical performance measures
SD: Standard deviation
SONIC: The Septuagenarains, Octogenarians, Nonagenarians Investigation with Centenarians study
SPPB: The Short Physical Performance Battery
TMIG-IC: The Tokyo Metropolitan Institute of Gerontology Index of Competence
TMIG-LISA: The Tokyo Metropolitan Institute of Gerontology-Longitudinal Interdisciplinary Study on Aging
TUG: The Timed up and go
